# Fisher vs. the Worms: Extraordinary Sex Ratios in Nematodes and the Mechanisms that Produce Them

**DOI:** 10.3390/cells10071793

**Published:** 2021-07-15

**Authors:** Justin Van Goor, Diane C. Shakes, Eric S. Haag

**Affiliations:** 1 Department of Biology, University of Maryland, College Park, MD 20742, USA; ehaag@umd.edu; 2 Department of Biology, William and Mary, Williamsburg, VA 23187, USA; dcshak@wm.edu

**Keywords:** sex ratio, sperm competition, meiosis, local mate competition, nematodes

## Abstract

Parker, Baker, and Smith provided the first robust theory explaining why anisogamy evolves in parallel in multicellular organisms. Anisogamy sets the stage for the emergence of separate sexes, and for another phenomenon with which Parker is associated: sperm competition. In outcrossing taxa with separate sexes, Fisher proposed that the sex ratio will tend towards unity in large, randomly mating populations due to a fitness advantage that accrues in individuals of the rarer sex. This creates a vast excess of sperm over that required to fertilize all available eggs, and intense competition as a result. However, small, inbred populations can experience selection for skewed sex ratios. This is widely appreciated in haplodiploid organisms, in which females can control the sex ratio behaviorally. In this review, we discuss recent research in nematodes that has characterized the mechanisms underlying highly skewed sex ratios in fully diploid systems. These include self-fertile hermaphroditism and the adaptive elimination of sperm competition factors, facultative parthenogenesis, non-Mendelian meiotic oddities involving the sex chromosomes, and environmental sex determination. By connecting sex ratio evolution and sperm biology in surprising ways, these phenomena link two “seminal” contributions of G. A. Parker.

## 1. Introduction

Theodosius Dobzhansky famously stated [1] that “nothing in biology makes sense except in the light of evolution”. Similarly, little in evolution makes sense except in the light of reproduction. Sexual reproduction via anisogamy has repeatedly arisen across the breadth of multicellular organisms [2], suggesting a central role for the evolutionary success of these taxa [3,4,5]. The Parker, Baker, and Smith model is based on the apparent need for substantial zygotic provisioning in multicellular organisms. When the viability of a zygote is disproportionately harmed by reductions in such provisioning, a bimodal distribution of fitness emerges. That is, both large and tiny gametes stably co-exist. Although both types can be made in a single hermaphroditic sex, many lineages evolved completely separate male and female sexes.

Following Darwin [6] and Düsing (1884) [7], the differential investment in gametes was understood to exert strong ecological pressure on the ability to find mates, requiring that there be functional population-level sex ratios over evolutionary time to ensure persistence. Fisher [8] recognized that this feature generally gave a fitness advantage to the rarer sex, driving the maintenance of a 1:1 sex ratio (though local deviations can be expected, see Trivers and Willard [9] and Orzack, et al. [10]). Genetic sex determination (GSD) and Mendelian genetics often reinforce this [11]. However, because most populations with equal numbers of males and females produce far more male gametes (i.e., sperm) than are required to fertilize all the female gametes (i.e., oocytes), such populations exhibit intense levels of male and/or sperm competition [12,13,14]. Thus, selection for the rarer sex in an obligately outcrossing system imposes a seemingly unavoidable cost of males (see also Haldane [15]), which reaches a full two-fold disadvantage if embryos can be produced without any sperm-derived resources [16]. Under the Fisherian model, the restriction of reproductive potential at a population level is a necessary consequence of obligate outcrossing.

Darwin was aware that many organisms do not exhibit a 1:1 sex ratio in nature, and notoriously left “the problem for future generations” [17]. Hamilton [18] understood that Fisher’s model assumed large populations with random mating (panmixia). However, many organisms deviate from this characterization and are instead characterized by subdivided population structures in which individuals mate in very small groups in isolated and often ephemeral patches. This difference fundamentally shifts the selection on sex ratio due to a suite of mutually reinforcing consequences. First, these closed systems prevent males from accessing females outside of the local group, so that grandparental fitness is much greater with a female-biased sex ratio. Second, the resulting local mate competition (LMC) generates higher-level (“group”) selection among populations due to differential success in colonizing subsequent patches [19,20,21,22,23,24]. Third, repeated inbreeding causes the genotypes of male and female mates to converge and purges deleterious genotypes [25,26]. The resulting lack of both competition between genetically distinct males and of inbreeding depression allows for selection on sex ratio to be driven by differences in inter-patch fecundity.

The evolution of female-biased sex ratios in response to local mate competition conditions is often facilitated by haplodiploidy, in which unfertilized eggs develop as haploid males, and all fertilized eggs develop into diploid females. This is typical of wasps (Hymenoptera) [27,28,29] but is also found in mites (Acari) [30]. Perhaps most famously, haplodiploidy and local mate competition have been connected to population structure in fig wasps. When a wasp “foundress” oviposts eggs into a fig lacking other eggs, she will produce a highly female-biased brood (roughly 5–10% males). However, when multiple foundresses oviposit into the same fig, the proportion of males drastically rises [31,32,33,34].

Organisms that have evolved uniparental reproductive modes can be viewed as the most extreme deviation from Fisherian sex ratios. Here, males are eliminated constitutively or facultatively through self-fertile hermaphroditism [35,36,37] or parthenogenesis [16,38]. In both plants [39,40] and invertebrates [41], such strategies are often associated with mate scarcity [42] and generally evolve from outcrossing ancestors. Both reproductive modes appear to exact costs on lineages utilizing them, such as reduced adaptive potential [43,44], the progressive accumulation of deleterious mutations known as Muller’s ratchet [45]; and, in parthenogenetic species, the prevention of effective meiotic recombination [46,47]. All of these consequences are associated with reduced speciation and/or an increased likelihood of extinction [48]. Nevertheless, uniparental reproductive modes are widespread, are not always evolutionary dead ends, and evolve repeatedly because they increase fitness compared to outcrossing relatives [49,50].

In some cases, non-Fisherian sex ratios may be adaptive, yet unattainable. If both sexes are diploid and determined by chromosomes at fertilization, and meiosis is unbiased and constrained by the complex machinery that implements it, then a 1:1 sex ratio is inevitable, even if sub-optimal [51,52]. Once established under a regime favoring equal sex ratios, such genetic sex determination (GSD) systems could be a phylogenetic constraint. This appears likely in lizards [53], frogs [54], and some deep-sea invertebrates [55].

Although GSD may constrain the ability of organisms to manipulate their sex ratios, the generation of a wide range of ratios can be enabled via environmental sex determination (ESD) [56]. Here, sex is dictated by a physiological response to external factors, such as temperature, salinity, nutrient availability, sunlight, or mate access [22]. ESD typically evolves from GSD [57]. ESD is favored when organisms have little control over their environments and is frequently accompanied by the loss of sexually dimorphic variants [58,59,60]. ESD has been studied extensively in reptiles [61,62], but has also been described for fish [63], plants [64], and many invertebrates [65]. Although well adapted by their ability to optimize sex ratios, ESD-dependent species are vulnerable to extreme environmental perturbances, including current trends in global climate change [66].

Based on the above, we can observe that great progress has been made in explaining *why* non-Fisherian sex ratios are maintained in some organisms. However, we are only beginning to understand *how* these reproductive modes evolved. What is known tends to come from disparate exemplar taxa (e.g., haplodiploid Hymenoptera with female-biased sex ratios, uniparental/clonal plant species, reptiles with ESD, etc.). The phylogenetic distance between these groups limits our comparative abilities, and with that our understanding of the mechanisms that enabled their evolution. The ideal taxon would present a broad diversity of reproductive modes (ranging from obligate outcrossing to parthenogenesis) within a recently diverged taxon (e.g., genus, family-level). We suggest that nematodes offer such a taxon. In this review we will showcase the diversity of mechanisms used to generate Fisherian and non-Fisherian sex ratios in nematodes. In highlighting recent advances, we hope to inspire others to explore that which is still unknown.

## 2. Nematoda as a Model Phylum for the Evolution of Sex Ratios

Nematoda is one of the most diverse and successful metazoan phyla [67,68,69]. Its estimated one-million-plus species [70,71] occupy nearly every described habitat on the planet [72]. While they are famously parasitic [73], we now understand that nematodes exist in a vast array of free-living [74], commensal [75,76], and even mutualistic [77,78] contexts that have undoubtedly influenced the development of entire ecosystems. Importantly here, nematodes vary greatly in reproductive mode and sex ratio. This variation has likely been a key to their ecological and evolutionary success. It also makes them a model for studying the evolution and maintenance of sex ratios.

Beyond their sheer diversity, many nematodes are well-suited for research. Nearly all are transparent, and many have short lifecycles and tolerance to laboratory culture [79]. Further, oogenesis occurs in a single file “assembly line” fashion, making the ordering of events unambiguous. The preeminent model nematode is *Caenorhabditis elegans*, the first multicellular organism to have its genome fully sequenced [80]. Over 50 other *Caenorhabditis* species have been formally described, with their genomes published [81]. Other nematodes from different orders and families have gained attention due to their parasitism of plants, insects, or mammals. Nematode genomes are relatively compact and heterochromatin-free (<250 mB); [82,83,84], spurring an international effort to sequence nearly 1000 species across the phylum’s diversity [85], with more following as sequencing efforts become more affordable.

Nematodes’ reproductive mode can vary over short timescales, often within genera [41,86]. There are even examples of sexual polyphenism within the same species [87]. Although the XX/XO sex determination system is found in *Caenorhabditis*, *Pristionchus*, and many other species, [88,89,90], an XX/XY GSD mechanism was likely ancestral and can still be found (or has subsequently re-evolved) in certain groups [91,92]. Loss of the Y as an essential feature of males allows XO males to be produced spontaneously in uniparental species through meiotic disjunction of the X chromosome in XX females and hermaphrodites [93], which has important implications for the evolution and maintenance of outcrossing and of sex ratios in nematode species.

### 2.1. Fisherian Nematodes

Gonochorism, the obligate outcrossing of separate male and female sexes, was likely the ancestral condition and remains the most common reproductive mode for extant nematodes [86,89,94]. When combined with GSD and Mendelian segregation sex chromosomes, gonochorism generally enforces a 1:1 sex ratio [41,95]. In accordance with Fisherian assumptions, obligately outcrossing male–female species often show very little linkage disequilibrium [96,97], indicating near-random mating across a large population. Gonochorism is common in both free-living [98] and (perhaps surprisingly) many parasitic [91,99] species.

The life histories of many free-living nematodes are marked by “boom-bust” cycles [74,100]; in which an individual arrives at a reproductive patch, often vectored by an Arthropod host with which it shares resources. From a few founders, the population rapidly expands until resources are exhausted. Worms then disperse to a new patch, typically as dauer larvae [101,102]. In many parasitic species, dauer larvae are the environmentally tough stage that initiates a new infection. In gonochoristic species this boom and bust lifecycle usually coexists with a 1:1 sex ratio, despite the presence of population subdivision [89,94]. Reliable colonization of a reproductive patch by both male and female individuals may be mediated by sheer numbers. The human parasite *Brugia malayi*, which causes lymphatic filiariasis, provides an extreme example. A single adult female floods the bloodstream of its host with a thousand tiny larvae per day, and sustains this for years [103]. The larvae become so numerous that the tiny blood meal of a mosquito, amounting to only a few microliters, is sufficient to take up multiple larvae and allow their transfer to a new host. After multiple bites by infected mosquitoes, a single human can harbor many genotypes.

### 2.2. Non-Fisherian Nematodes

Although many nematode species exhibit life histories that appear to be consistent with the pressure of local mate competition, only a modest subset has been found to produce female-biased sex ratios. Haplodiploidy among nematodes is uncommon and has only been described in the order Oxyurida (pinworms). As in other haplodiploid animals, this shift has been proposed to be an adaptive mechanism for ensuring female-biased sex ratios in subdivided host patches [104]. There are other (non-haplodiploid) mechanisms through which nematodes respond to local mate competition, and these will be the focus of the rest of this review.

The local sex ratio can be strongly influenced by factors other than sex determination. Within the genus of insect-parasites *Steinernema*, sex-biased foraging strategies interact with host choice to produce local male-biased (in early-attacking individuals) [105] or female-biased broods (late-attacking and overwintering). This temporal shift interacts with male–male fighting for access to females [106] and an apparently intrinsically female-biased embryo pool to generate unequal sex ratios across infected hosts [107]. Similarly, female-biased sex ratios have been tied to differential transmission ability and longevity between the sexes in gonochoristic mammalian parasitic nematodes [108,109]. Finally, differential survivorship, as well as subdivision, has been proposed to explain population-level fluctuations in the sex ratio, arriving at general female-bias in obligately outcrossing members of the cold- and desiccation-tolerant genus *Panagrolaimus* [110,111]. The specific cellular and physiological bases of sex determination and differential mortality in these species remain largely unknown.

Species that experience pressure for female-biased sex ratios can ultimately evolve reproductive systems that are intrinsically female-biased. These can be based on non-Mendelian meiotic phenomena or environmental sex determination (both discussed further below), as well as uniparental reproductive modes (either self-fertility or parthenogenesis). The evolution of self-fertile hermaphrodites from outcrossing female ancestors has been studied extensively in *Caenorhabditis* [41,86,112] and *Pristionchus* [113], where independent origins of self-fertility have been observed. Future research may also focus on *Panagrolaimus*, where outcrossers, hermaphrodites, and parthenogenetic taxa are found in the same genus [111,114].

The shift to uniparental reproduction has important organismal consequences. Selfing species exhibit degraded reproductive behavior [115] and can experience the substantial loss of genome size and gene content [116,117,118]. The latter is driven by a combination of relaxed purifying selection, positive selection (see below), and sex-biased segregation of large insertion–deletion polymorphisms during male meiosis, which we term indel segregation distortion (ISD) [119,120]. Although ISD occurs in both gonochoristic and selfing *Caenorhabditis*, its existence is obscured in the former, as every embryo has two parents. When both mating and selfing co-occur, however, the tendency for larger alleles to be passed to sons deterministically increases their rate of loss because of the greater variance in male reproductive success [11].

**Figure 1:**
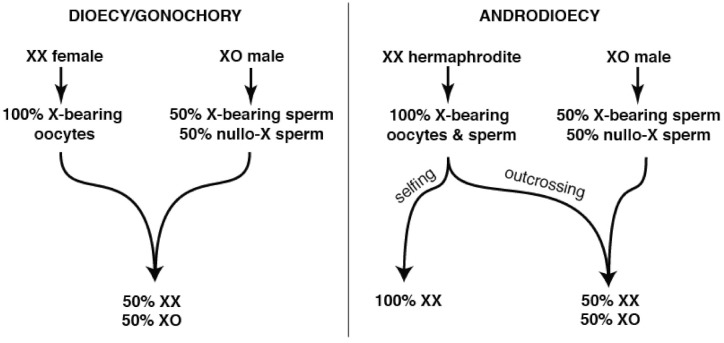
Interaction of XX/XO sex determination and self-fertility. (**Left**): XX/XO sex determination is found in *Caenorhabditis, Pristionchus*, and many other less-studied nematodes. When combined with obligate outcrossing, it reinforces the 1:1 male:female ratio. (**Right**): When XX females evolve self-fertility, purely Mendelian dynamics render the sex ratio dependent upon the extent of selfing vs. outcrossing with XO males. Pure selfing produces almost exclusively XX hermaphrodites, with very rare XO males produced spontaneously through aneuploidy. However, complete suppression of hermaphrodite self-fertility by males recreates the 1:1 ratio of the outcrossing ancestors. Such suppression requires retention of pre- and post-copulatory traits that promote outcrossing, especially in males.

Unsurprisingly, many of the genes lost in the wake of self-fertility include those required for successful male reproductive competitiveness [118]. However, this loss is unlikely to occur wholly through relaxed purifying selection and genetic drift. In XX/XO species with the androdioecious (hermaphrodite/male) mating system, the extent of male sperm precedence (relative to self-sperm) is a key determiner of the population’s sex ratio (Figure 1).

Frequent selfing results in populations that tolerate high levels of inbreeding [25,121]. This allows hermaphrodites to produce comparable numbers of offspring, and greatly increased numbers of grand offspring, if they forgo outcrossing entirely. However, uniparental species require periodic outcrossing to counter new deleterious mutations and to pass on essential adaptations [44,47]. This ongoing value of outcrossing counters the tendency to fix spontaneous male-lethal or male-sterile mutations, which forward genetic screens in *C. elegans* suggest are otherwise easily produced [122,123].

## 3. Mechanisms Producing Non-Fisherian Sex Ratios

A growing number of nematode species are known to deviate from equal sex ratios. Each appears to represent adaptive tuning to specific ecological conditions. Female-biased sex ratios are mediated in some cases by self-fertility and associated impacts on male function and in other cases by “meiotic scandals” that violate conventional expectations for GSD systems. In some species, non-Mendelian processes alternate with more conventional ones to form complex heterogonic life cycles that unfold over multiple generations. In a few species, unequal sex ratios are achieved through environmental sex determination (ESD) that is established well after fertilization. These variations highlight the ways in which the molecular and cell biology of meiosis and fertilization can respond to selection imposed by deviations from idealized Fisherian populations.

### 3.1. Path 1: Maintenance of GSD, but with Self-Sperm (Androdioecy)

Organisms that evolve self-fertility through hermaphroditism are expected to experience higher fitness than their obligately outcrossing relatives in environments of mate scarcity and/or population subdivision [37,50]. Although many free-living nematodes face these environmental conditions, selfing may not always evolve. First, the complex developmental adaptations required to evolve a hermaphrodite gonad may simply fail to appear. Second, even when they do appear, inbreeding depression may doom an incipient selfing lineage before it can stabilize. However, if both of these hurdles are overcome, self-fertility eliminates the cost of males in favor of highly productive hermaphrodite-rich populations [124,125]. Indeed, natural populations of selfing *Caenorhabditis* [126], *Pristionchus* [113], and *Bursaphelenchus* [127] are composed almost entirely of hermaphrodites. Thus, while males were essential in their gonochoristic ancestors [86] and likely remain important in order to clear deleterious mutations and enable adaptation [44,47], they are generally rare.

The evolution of hermaphroditism requires a developmental novelty: the production of fertilization-competent sperm in an otherwise female body [112]. In nematodes, sperm have at least three roles. Most obviously, they provide the haploid genetic complement to the oocyte and trigger embryonic development [128]. In addition, sperm are also required for egg activation and ovulation [129,130,131], and sperm provide the centrosome for the zygote [132,133]. In *C. elegans* the sperm also provides SPE-11, a novel protein necessary for successful embryogenesis [134] and for the prevention of polyspermy [135].

Fertilization takes place within the spermatheca for both outcrossing and hermaphroditic nematodes, but the sperm’s route to this site differs. During mating, outcrossing sperm and seminal fluid exit through the male reproductive tract, and the initially immotile spermatids are activated within the uterus [136]. The sperm then migrate “up” to the spermathecae, where they await ovulations. In contrast, hermaphrodites’ self-sperm are produced in the otherwise oogenic germ line and migrate “down” to the spermatheca before the first ovulation [137,138]. Notably, both male and hermaphrodite sperm are frequently swept into the uterus by ovulating oocytes but have the ability to repeatedly migrate back to the spermatheca for additional fertilization opportunities. Hermaphrodite self-sperm evolved the ability to activate in the absence of male gonadal signals [139], though it is possible this may have evolved from an intermediate state of mating-dependent trans-activation [140]. Although important aspects of how XX spermatogenesis evolves remain unclear, essential components have now been identified in both *C. elegans* and *C. briggsae*. In *C. elegans*, FOG-2 and GLD-1 are essential post-transcriptional, germline-specific co-repressors of the global female-promoting gene *tra-2* [141,142,143]. Parallel genetic investigations in *C. briggsae* have shown that the convergently evolved hermaphrodites differ in both species-specific genes [144,145] and in the divergent context-dependent roles of conserved factors [146,147,148]. The details of germline sex determination and its evolution have been reviewed recently elsewhere [140,149].

**Figure 2:**
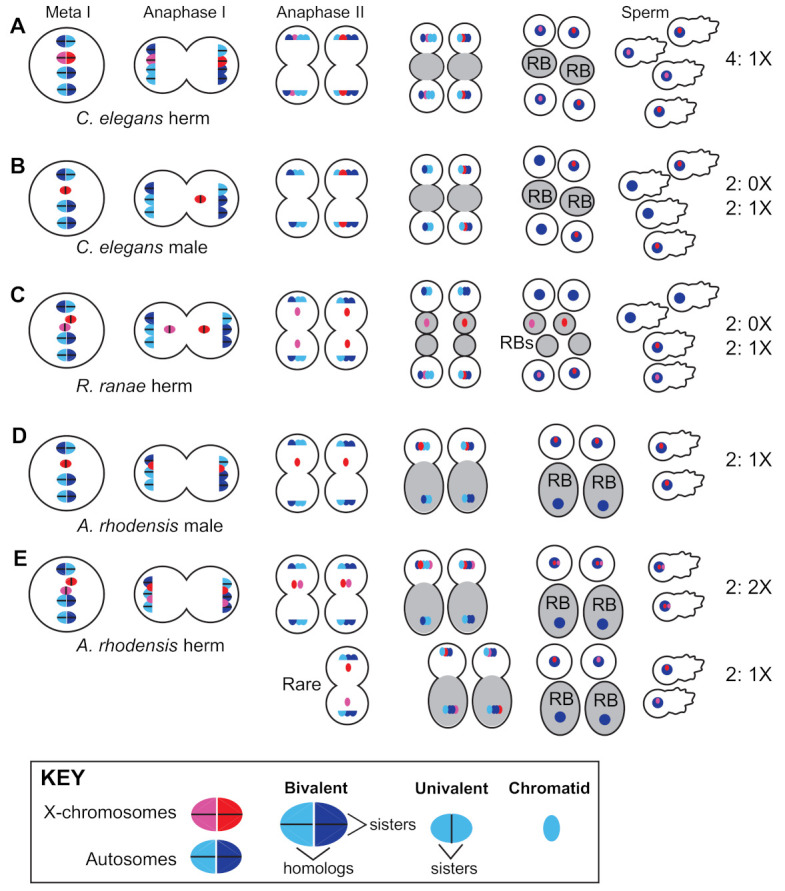
Mendelian and non-Mendelian patterns of X-chromosome segregation during nematode spermatogenesis. (**A**) In the meiotically dividing spermatocytes of XX *C. elegans* hermaphrodites, the homologous X chromosomes (red/pink) pair, recombine, and segregate in a manner indistinguishable from the autosomes (blue). The products of spermatogenesis are four 1X sperm. An important but unusual feature of nematode spermatogenesis is that a residual body (gray) forms between the haploid sperm immediately following anaphase II, rather than after an extended, post-meiotic process of sperm differentiation. In addition, the sperm locomote using a pseudopod rather than a flagellum. (**B**) Meiotically dividing spermatocytes of *C. elegans* males have a single X chromosome (an unpaired univalent) which lags during anaphase I and ultimately segregates to one of the two secondary spermatocytes. Spermatogenesis ultimately yields two 1X and two 0X sperm. (**C**) In one variation, self-fertilizing XX *Rhabdias ranae* hermaphrodites can produce a limited number of 0X sperm and thus male progeny because unpaired X chromosomes lag during both meiotic divisions and lagging X chromatids can be left behind in the residual body following anaphase II. (**D**) In spermatocytes of *Auanema rhodensis* males, the single X chromosome splits into sister chromatids during meiosis I. During meiosis II, the X-bearing chromosome complement segregates with the functional sperm components, whereas the non-X bearing chromosome complement segregates to the residual body. Thus, the product of male spermatogenesis is two 1X sperm and two DNA-containing residual bodies. (**E**) In spermatocytes of *A. rhodensis* hermaphrodites, the homologous X chromosomes fail to pair and instead split into sister chromatids during the first meiotic division. During the second division, the non-sister X chromatids typically segregate with the functional sperm components to generate 2X sperm. Young hermaphrodites produce a small number of 1X sperm, presumably via the indicated mechanism (rare), which enables the production of male offspring early in the brood.

Hermaphrodites of the *Caenorhabditis* and *Pristionchus* genera employ standard meiotic sex chromosome segregation in both oocytes and sperm. XX hermaphrodites produce 100% X-bearing sperm [150] (Figure 2A) and selfed zygotes are generally XX, like their mothers. In XO male spermatogenesis, the un-paired X lags during anaphase I before ultimately segregating to one of two secondary spermatocytes. Males thus produce equal numbers of X-bearing and 0X sperm [151] (Figure 2B), and the more male sperm are used by hermaphrodites, the closer to 50:50 the sex ratio becomes (Figure 1).

However, because males are both optional for reproduction and relatively rare, selfing greatly reduces the strength of sexual selection, including sperm competition [95]. Over time, divergent levels of sperm competition lead to dramatic effects in interspecific crosses between outcrossing males and hermaphrodites. Aggressive, interspecific-male sperm exhibit invasive (and often lethal) behavior in hermaphrodites that conspecific outcrossing females readily resist [152]. Because the most likely inter-species cross between closely related gonochoristic and androdioecious species is this same hermaphrodite X male situation, invasive sperm represent a bizarre form of post-copulatory, pre-zygotic reproductive isolation.

Our understanding of the cellular, molecular, and genic factors that modulate sperm competition and inter-species sperm invasion is limited but growing. *Panagrellus* sperm appear capable of forming sperm-chains within the uterus [153]. Although the competitive advantage of this adaptation remains unclear, it is reminiscent of cooperative sperm aggregations in *Peromyscus* mice [154]. Male sperm in *Caenorhabditis elegans* are larger in size and outcompete conspecific hermaphroditic sperm [155,156], highlighting key differences between the two sperm types. Additional factors beyond size are just starting to be explored. Genetic screens identified a pseudokinase COMP-1 within the cell body of the sperm that is essential for effective sperm competition [157]. Genomic comparisons of species within the *Caenorhabditis* genus identified the sperm surface glycoprotein male secreted short (MSS), which has been lost in all selfing species and is essential for successful sperm competition in outcrossing *Caenorhabditis* [118]. Remarkably, restoration of *mss* to self-fertile *C. briggsae* via transgenes is sufficient to enhance the sperm precedence of males relative to both *mss-* males and to self-sperm.

If *mss* can improve the fitness of *C. briggsae* males, why was it lost in parallel in all selfing species? While relaxed sexual selection is intuitively appealing, both theory and experiments suggest that this is inadequate [124]. Experimental evolution and modeling both indicate that functional *mss* alleles will invade at the expense of *mss*-null alleles whenever males are present in an androdioecious population. However, in increasing the number of fertilizations by 0X sperm, this will shift the sex ratio to be less hermaphrodite-biased (Figure 1). In genetically homogenous populations that lack inbreeding depression (as in *Caenorhabditis elegans* and *C. briggsae*, which may actually experience outbreeding depression [158]), *mss*-null patches quickly out-reproduce mixed populations due to their more hermaphrodite-biased sex ratios [124]. This suggests that *mss* is actually pushed out of the genome by local mate competition-like inter-demic selection on subdivided hermaphrodite populations. By reducing, but not eliminating, the use of outcross sperm, *mss* loss allows selfing species to fully exploit their growth advantage, while still enabling occasional outcrossing.

Presumably, many sperm and male competition mechanisms have yet to be discovered within nematodes. Their transparency allows us to observe this competition in vivo, making further inquiries highly enticing.

### 3.2. Path 2: Maintain GSD, but Enrich for Females with Facultative Parthenogenesis

Parthenogenesis marks a complete departure from Fisherian expectations, and theory suggests that it can be an extremely successful reproductive mode [16]. There are multiple origins of parthenogenesis in Nematoda [43], generally mediated by deviations from Mendelian chromosome segregation during meiosis [159]. In *Diploscapter pachys* parthenogenesis is obligate, as meiosis I does not occur and key meiosis genes were lost from the genome [160]. Intriguingly, these changes were accompanied by fusion of its chromosomes into a single unichromosome and the locking in of substantial heterozygosity. Other species within this genus (e.g., *Diploscapter coronatus*) have lost some meiotic genes but retained other aspects of meiosis [161]. However, the adaptive constraints imposed by asexual reproduction make obligate parthenogenesis rare [162], with facultative parthenogenesis being only slightly more common [163]. Furthermore, parthenogenesis is often paired with sexual modes through heterogony, in which reproductive modes vary across generations, as in the parasite *Strongyloides ratti* [164,165].

For some species, parthenogenic development is pseudogamous. This means a sperm is still required to launch development, even though its genome is not propagated. Sperm may be required for activation alone, or for both embryo activation and provision of the first centrosome. Sperm typically comes from males of a related XX/XO or XX/XY species, as can be seen in laboratory hybrid crosses between *Caenorhabditis becei* males and *C. nouraguensis* females [166]. However, for the XX/XY genus *Mesorhabditis* these sperm are restricted to males of the same species, as attempted interspecies crosses are thwarted by blocks prior to sperm–oocyte fusion or incompatibilities with the paternal centrosome [167]. Natural populations of *Mesorhabditis belari* thus cannot be exclusively female, but are highly female-biased (91% XX female and 9% XY male).

**Figure 3:**
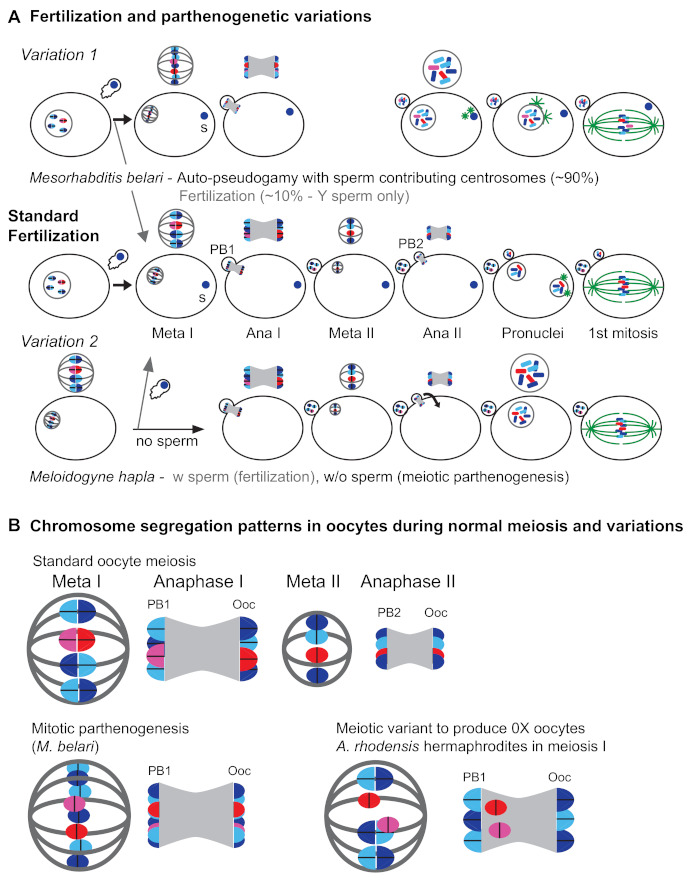
Oocyte meiosis during normal fertilization and parthenogenetic variations. (**A**) Cell-level view (**B**) Enlarged view of chromosomes (blue—autosomes, red/pink—X) and meiotic spindles (gray). (**A**—middle) During standard fertilization, the meiotic divisions of the oocyte chromosomes occur only after sperm entry. Oocyte meiosis is associated with acentriolar spindles and during each division, half of the chromosomes are segregated during a highly asymmetric division to a small polar body (PB1, PB2). Homologs separate during the first (reductive) meiotic division, and sister chromatids separate during the second meiotic division (enlarged in **B**). During these meiotic divisions, the sperm chromatin(s) remains as a highly condensed, haploid chromatin mass. Following the second meiotic division, the haploid genomes of both oocyte and sperm acquire pronuclear envelopes and undergo a round of DNA synthesis before coming together for the first mitotic division of the embryo. The microtubule asters (green) are formed on sperm-supplied centrosomes. (**A**—Variation 1) In *Mesorhabditis belari*, most sperm enter but their genetic material remains as a tight chromatin mass(es) that does not contribute to the embryo. Instead, the oocyte chromosomes undergo a single, mitotic-like spindle with unpaired homologs (enlarged in **B**) to generate offspring that are maternal clones but with sperm-derived centrosomes. When sperm do participate in standard fertilization (gray arrow), the progeny are male, since only Y-bearing sperm participate in fertilization events. (**A**—Variation 2) In *Meloidogyne hapla*, normal fertilization occurs when sperm are present (gray arrow). In the absence of sperm, the oocyte chromosomes undergo the normal sequence of meiotic divisions, but chromosomes from the second division rejoin their sister chromatids (arrow) to generate an embryo with half the genetic diversity of their mother. (**B**) In *Auanema rhodensis* hermaphrodites, the homologous X chromosomes (red/pink) fail to pair and instead line up as univalents during metaphase I. During anaphase I, both Xs segregate to the polar body, which ultimately results in the production of 0X oocytes.

Because male sperm enter the oocyte yet never contribute to the genetic complement of the female *M. belari* embryo, the species exists as a set of all-female maternal clones [168]. However, Y-bearing male sperm do occasionally induce complete oocyte meiosis and nuclear fusion, and it is these rare cases that produce the next set of XY males (Figure 3).

A different system is used by the plant-parasitic root knot nematode *Meloidogyne hapla. M. hapla* is an XX/XO species that undergoes facultative parthenogenesis while in association with its host. If XO males are present, the male sperm fuse with the oocyte and normal fertilization ensues, resulting in a 1:1 ratio of males to females [169]. However, in the absence of sperm, the sister chromatids from the second meiotic division will reunite into a single pronucleus to form XX daughters with half the genetic diversity of their mother [170]. This system is only possible because these oocytes have bypassed the need for sperm to mediate egg activation, and the oocyte retains centrosomes. In both *M. belari* and *M. hapla*, plasticity is facilitated by the general tendency of nematode oocytes to perform both meiotic divisions after sperm entry.

### 3.3. Path 3: Combine GSD with Non-Mendelian Sex Chromosome Segregation

The potential for XX hermaphrodites to produce spontaneous XO offspring via meiotic modification is likely an important facilitator of exceptional sexual modes in nematodes. For example, the frog parasite *Rhabdias ranae* has both an infective, host-seeking male–female phase followed by a reproductive hermaphroditic phase while inside of the lungs of its host [171]. Here, the XX hermaphrodite produces not only XX females, but also XO males. The latter are formed when X-chromatids lag during anaphase II of spermatogenesis and are subsequently lost in a residual body, resulting in the production of 0X sperm (Figure 2C) [172]. However, since this occurs in only some X-chromatids, most of the self-progeny are XX, and thus the offspring are female-biased [173].

Species in the genus *Auanema* present an especially intriguing suite of oddities related to sexual mode and sex ratio. They are naturally trioecious, with XO males, XX females, and XX hermaphrodites all found in a single population [174,175]. They likely arose from a conventional XX/XO outcrossing system [90], allowing several derived features to be recognized unambiguously. Though retaining GSD, *Auanema* produces XX-biased sex ratios (as both females and hermaphrodites). Unlike *C. elegans*, *Auanema* hermaphrodites produce sperm and oocytes simultaneously via spermatogonial stem cells, making them an appealing comparative satellite organism in the study of gametogenesis [176].

In *Auanema rhodensis*, although meiosis in XX females almost always follows standard Mendelian rules, X chromosome segregation in hermaphrodites and males do not [177,178]. Non-Mendelian modes of X chromosome segregation are seen in the spermatogenesis of both males and hermaphrodites (Figure 2D,E) and hermaphrodite oogenesis (Figure 3B). In males, spermatogenesis results in the formation of two X-bearing sperm and two residual bodies containing the full non-X-bearing chromosomal complement [177,179]. During hermaphrodite spermatogenesis, both Xs divide into sister chromatids during meiosis I. Then, during meiosis II, the two non-sister chromatids are segregated to the 2X functional sperm, whereas the residual bodies receive the non-X genetic complement [178]. This non-Mendelian segregation of the X chromosome during hermaphrodite spermatogenesis is accompanied by a lack of X-chromosome recombination during the meiotic prophase. To complement their 2X sperm, hermaphrodites produce 0X oocytes by segregating both X chromosomes to the polar body during meiosis I [178].

These gamete- and sex-specific patterns of X chromosome segregation impact subsequent generations in *A. rhodensis* in interesting ways. Since males (1X sperm) have a strong preference for mating with females (1X oocytes) [174], the progeny of such crosses are almost exclusively XX hermaphrodites. On the other hand, rare male progeny from male–female crosses carry the paternal X, revealing that female meiosis can occasionally generate 0X oocytes [178]. Furthermore, although self-fertilizing *A. rhodensis* hermaphrodites produce mostly hermaphrodite progeny, they routinely produce males and females early in their broods [174], an adaptation which hastens their production of grandchildren since the sexual maturation of their hermaphroditic offspring is delayed by their obligate passage through a dauer larval stage.

The cellular basis of the meiotic scandals of *A. rhodensis* has been investigated in some detail. The spermatocytes are unusually small, which may be why the spermatocyte meiosis in both males and hermaphrodites yields only two rather than the normal four sperm [179]. Their unusual pattern of X chromosome segregation combined with the functional equivalent of sperm polar bodies supports dramatically skewed sex ratios during both male and hermaphrodite spermatogenesis. In hermaphrodites, altered patterns of X chromosome segregation depend on the absence of meiotic recombination, specifically between the X chromosomes but not the autosomes. Exactly how this is regulated remains unclear, but *C. elegans* mutants with defects predominantly in X-chromosome segregation may provide clues [180,181,182]. Other clues may be provided by the more extreme case of mitotic parthenogenesis (Figure 3B), when the meiotic pairing of all homologs, both X-chromosomes and autosomes, fails to occur [183]. During mitotic parthenogenesis, the univalents undergo a single mitotic division to form one polar body and the progeny are genetic clones of their mother [184].

### 3.4. Path 4: Abandon GSD and Use Environmental Sex Determination

**Figure 4:**
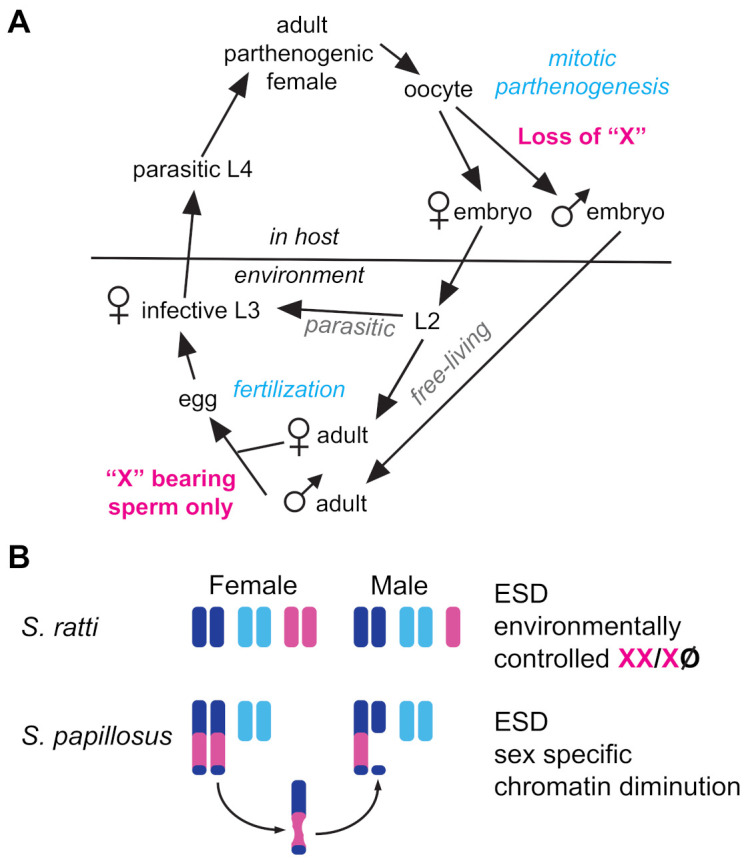
Life cycle and Chromosomes in *Strongyloides*. (**A**) Life cycle of *S. ratti* and *S. papillosus*. Adult XX females within hosts produce three types of progeny via mitotic parthenogenesis: (1) eggs that develop directly into female parasitic morphs, (2) eggs that become sexual free-living females, and (3) eggs that become free-living sexual males through the loss of one X chromosome. Males and females produce offspring through fertilization but these only develop into parasitic females. (**B**) Like most *Strongyloides* species, *S. ratti* females have a diploid chromosome number of six, and *S. ratti* males have a diploid number of five. To form *S. ratti* males, one X is lost during mitotic parthenogenesis by a yet-unknown mechanism. *S. papillosus* have chromosome number that reflects an insertion/fusion of the X chromosome into an autosome, forming a long chromosome. During mitotic parthenogenesis of some oocytes, the X region of one of the two long chromosomes is lost via chromatin diminution. Such embryos develop into males.

A growing number of nematodes have been recognized to deviate from equal sex ratios via environmental sex determination (ESD). As with other taxa, ESD in nematodes evolves from ancestral GSD mechanisms [185] in response to similar environmental pressures [186]. Nematode ESD mechanisms described to date generally result in female-biased sex ratios [187]. In intestinal parasites of the genus *Strongyloides*, ESD relies on manipulation of ancient GSD machinery. Parasitic females are parthenogenic, but they produce both parasitic and free-living female clones through mitotic parthenogenesis (Figure 4A). They also produce free-living XO males when mitotic parthenogenesis is accompanied by the loss of a single X chromosome [188,189] (Figure 4A).

Exactly how this X chromosome is lost in *S. ratti* remains unclear, but in *S. papillosus*, the production of males involves the diminution of X-derived chromatin within an autosome–X fusion chromosome [190]. A second mystery presented by *Strongyloides* is how the free-living males sire exclusively female progeny, rather than a 1:1 mix of male and female progeny (Figure 4A). This may be due to non-Mendelian meiosis, as described above.

Environment comes into play in determining whether *Strongyloides* parasitic females produce mostly parasitic females or free-living morphs. In addition, the production of males increases with higher temperatures, increased population densities, and a more robust immune response by the host [165]. In *C. elegans*, the developmental decision of whether a L1 larva will develop directly into a reproductive adult or instead develop into a dauer larvae is dictated by environmental conditions and sensed through insulin and TGFb-like pathways [191,192]. Remarkably, the steroidal hormone Δ7-dafachronic acid, which functions downstream of these signals, not only suppresses dauer formation in both *C. elegans* and *Pristionchus pacificus*, but when the progeny of free-living *S. papillosus* larvae were grown on plates with Δ7-DA, they developed directly into free-living sexual females rather than dauer-like infective larvae [193]. Notably, they did not develop into males, suggesting that the mechanism by which free-living males produce only functionally X-bearing sperm remained unaffected.

The sex ratios of some nematode species are dictated strictly by ambient environmental conditions. For example, bacterivorous nematodes of estuaries produce significantly female-biased local sex ratios in response to high temperature or salinity. However, this may be due to a difference in environmental tolerances (e.g., survival) between the sexes rather than ESD [194]. Other nematode groups, notably plant parasitic species, have been described to allocate sex ratios based on nutrient availability [57]. For example, members of the *Melodogyne* and *Heterodera* genera produce female-biased sex ratios when nutrient availability is high and produce significantly more males under adverse environmental conditions [195,196]. Interestingly, while *Pristionchus* nematodes employ a conventional XX/XO GSD mechanism, the type of nutrients available within their local environment biases mouthpart polyphenisms that dictate whether they are carnivores, bacterivores, or fungivores [87,100].

When a species lives and breeds in isolated and ephemeral patches founded by a few individuals, local mate competition is expected to select for strongly female-biased sex ratios. In nematodes, these conditions are common in both free-living bacterivores and in parasitic species. Perhaps most famously, the mosquito-parasitic family Mermithidae generates female-biased sex ratios that vary by density of infection [197]. Specifically, sex is determined in a “female-first” fashion: females are most prevalent in small founding populations, but when populations become larger they become male-biased [198,199]. Similarly, nematode associates of fig wasps (*Parasitodiplogaster* and *Ficophagus*) have been observed to produce “overly precise” female-biased sex ratios (roughly 30–35% male), regardless of mating pool size [200,201]. This strategy ensures consistent reproductive success in the face of routinely low mating pool sizes, which would not be possible with GSD mediated by a Mendelian assortment of sex chromosomes. Although the underlying cellular mechanisms remain to be discovered, the phenomenon cannot be explained by differential larval mortality in the host. ESD acting between the infective larvae and adult stages (as in mermithids) is thus the most plausible explanation. While this density-dependent, female-biased mechanism may be more widespread than previously appreciated (observations here across three divergent orders of Nematoda), the molecular mechanisms providing this ability remain unknown.

### 3.5. Intersectionality—Auanema and Beyond

Trioecy in *Auanema* is likely maintained through persistent environmental stress and perturbation, which pressures dauer formation and dispersal to create new reproductive patches as hermaphrodites [202]. Consistent with this, the genus includes extremophiles with high arsenic resistance [203]. The switch between the alternative XX sexes (hermaphrodite vs. female) is itself a form of ESD. For all *Auanema* species, passage through the dauer stage is necessary and sufficient to specify hermaphrodite fate [175,204]. However, *Auanema* species differ in regards to when the associated female/hermaphrodite decision is made. In *A. rhodensis*, XX larvae commit to hermaphrodite fate by the L1 stage and pass through a dauer larval stage even when food is abundant and density is low [205]. The tendency for an XX offspring to develop as a female is influenced by the sex and age of the mother, as selfing hermaphrodites produce most of their female progeny early in the brood [174]. Nevertheless, the developmental decision of XX animals to become females or hermaphrodites remains plastic through the first larval stage and can be experimentally modulated by the addition of either dauer pheromone inhibitors or conditioned media from densely populated plates [204].

In *Auanema freiburgensis*, the ratio of hermaphrodite and female progeny depends solely on the condition of the hermaphroditic parent [175,206]. Hermaphrodites grown at low density produce only females, whereas those grown at high density produce only hermaphrodites [206]. These ratios can be readily and reversibly modulated within an individual hermaphroditic parent by treating her with either conditioned media or pharmacologically manipulating factors downstream of the dauer signaling pathway. In contrast to *A. rhodensis*, direct treatment of the larvae has no effect. Over the short history of any *Auanema* population, the proportion of each sex is likely to be highly dynamic. However, even closely related species differ in regard to the points of developmental plasticity and whether, as in the case of *A. rhodensis*, they are continuously producing some proportion of dauer/hermaphrodites in anticipation of degrading environmental conditions.

Many nematodes employ a mix of GSD and ESD, which exemplifies the opportunistic nature of reproductive evolution. There are likely many ways to flip the sex of an offspring, and these can act at or below the level of sex chromosome dosage. It is likely that more examples will be reported in the near future, as the work reviewed here may inspire researchers to follow up on the many possibilities.

## 4. Conclusions

The phylum Nematoda is composed of an extremely large number of species found across ecologically diverse habitats; however, we are only beginning to scratch the surface of its vast underlying diversity. Shockingly, our current estimate of over one million true species [70,71] is likely to be an underestimate. Haldane [207] suggested that every organism is associated with at least one parasite during its life history. Growing evidence suggests that at least one of these parasites may be a nematode in identity, especially among invertebrate communities. Furthermore, it is becoming more apparent that nematode associates are not always parasitic in nature but may also exist as commensals or even provide mutualistic services. Given that nematodes exist in nearly every described habitat [72], it is therefore possible that every eukaryotic organism is associated with at least one nematode during its life history in some ecological context.

As outlined in this review, nematodes exhibit an immense diversity in reproductive modes, which contribute to the evolution of Fisherian and non-Fisherian sex ratios alike. The success of these reproductive modes and the associated sex ratio adjustments have unquestionably contributed to the evolutionary success of the nematode phylum as a whole. Therefore, further evaluation of this reproductive mode and sex ratio allocation plasticity are worthwhile endeavors for future study. However, despite our current knowledge of the diverse mechanisms responsible for generating extreme non-Fisherian sex ratios, we are only just beginning to understand the true scope of this diversity. Undoubtedly there are examples and mechanisms yet to be discovered that will extend every topic presented here, and beyond.

Unsurprisingly, many puzzles regarding sex ratio adjustment in Nematoda are already evident. A clear example is how nematodes thrive in what we humans would regard as extreme habitats. Nematodes exist in the Arctic/Antarctic [208], as well as in the deep sea and in and around hydrothermal vents [209]. What adaptations have allowed these groups to flourish over time, and how do they reproduce? How do nematodes that must disperse over vast and challenging land/seascapes ensure their reproductive success? Why has uniparental reproduction not evolved in cases in which a life history would seem to benefit from it?

There are a number of challenges impeding the resolution of these outstanding puzzles. Some of the nematodes described here are extremely difficult to collect and may be impossible to culture for future experimentation. This is particularly true for obligate parasites [210], making their study difficult in the lab. Further, the stereotyped body plan of most nematode species makes them difficult to identify based on morphology alone. Genomic sequencing efforts will remain an invaluable resource for the identification of species and to reveal the true diversity that exists in the phylum. This will likely increase as next-generation and third-generation technologies become more affordable and sophisticated [211]. Finally, nematode researchers can be isolated due to their specific research questions, focal taxa, funding sources, favored publication outlets, and technical expertise. Greater collaboration between these subgroups will clearly be fruitful.

Despite these challenges, the work reviewed here amply demonstrates that nematodes have already begun to reveal their tremendous potential for linking reproductive phenomena at different levels of biological organization. We can expect much more in the near future. The diversity of non-parasitic species that can be grown on standard media in the laboratory is ever-expanding, along with more information about their natural ecological settings. Their transparency and similar basic anatomy make microscopy and working with multiple species relatively straightforward. Methods for genetic manipulation are rapidly advancing, with CRISPR/Cas9-based editing likely to be universally applicable. These assets set the stage for rich integrative research programs that can unite molecular, cellular, organismal, and ecological elements into a whole.

## Data Availability

Not applicable.
